# Lateral trochlear lengthening osteotomy

**DOI:** 10.1007/s00402-020-03736-5

**Published:** 2021-02-24

**Authors:** Petros Ismailidis, Christian Egloff, Corina Nüesch, Annegret Mündermann, Geert Pagenstert

**Affiliations:** 1 Department of Orthopaedics and Traumatology, University Hospital of Basel, Spitalstrasse 21, 4031 Basel, Switzerland; 2 Department of Clinical Research, University of Basel, Schanzenstrasse 55, 4056 Basel, Switzerland; 3 Department of Spine Surgery, University Hospital of Basel, Spitalstrasse 21, 4031 Basel, Switzerland; 4 Clarahof, Clinic of Orthopaedic Surgery, Knee Institute Basel, Clarahofweg 19a, 4058 Basel, Switzerland

**Keywords:** Trochlear osteotomy, Trochlear lengthening, Patella instability, Patellofemoral pain

## Abstract

**Introduction:**

The purpose of this study was to describe the indications and technical aspects as well as evaluate the clinical and functional outcome of lengthening osteotomy of the lateral trochlear ridge in patients with patellofemoral pain and/or patella instability and presence of trochlear dysplasia Dejour type A or lack of Dejour type dysplasia and short lateral articular trochlea.

**Materials and methods:**

Six consecutive adult patients were treated with a lateral trochlear lengthening osteotomy. Five patients received a concomitant medial patellofemoral ligament reconstruction. Three patients had prior patella stabilization surgeries. Trochlea dysplasia (Dejour classification), Caton–Dechamps index, tibial tubercle trochlear groove (TT-TG) distance, patellar tilt and lateral condylar index were measured in preoperative MRIs. Clinical and functional evaluation included the Kujala Anterior Knee Pain Scale, the Lysholm Knee Score, the Tegner Activity Score, a subjective evaluation form and isokinetic muscle strength.

**Results:**

Four patients had a Dejour type A dysplasia, two patients had no dysplasia. The mean (range) Caton-Dechamps index was 1.09 (0.95–1.16), TT-TG distance 10.9 mm (9.2–15.6 mm), patellar tilt 15° (4–32°) and lateral condylar index 82.0% (74–90%). One patient was lost to follow up, all others were followed for 2–5 years. All patients were satisfied with the clinical outcome. The Lysholm score increased from 55 (37–79) to 89 (76–100), the Tegner activity score from 3.6 (2–6) to 5.6 (5–7). The Kujala score postoperative was 90 (75–96). Some but not all patients had full strength recovery compared to the contralateral leg.

**Conclusion:**

We recommend measuring the lateral condylar index and considering the indication of a lateral trochlear lengthening osteotomy as an additional or isolated procedure in selected patients with trochlear dysplasia Dejour type A or lack of dysplasia and short lateral articular trochlea depending on the extent of the patellar instability.

**Level of evidence:**

Level IV, Case Series.

**Trial registration number:**

NCT04378491, clinicaltrials.gov, May 7, 2020.

## Introduction

Trochlear dysplasia has been recognized as one of the main reasons leading to patellofemoral instability and is found in 85% of patients suffering from recurrent instability [1]. Trochlear dysplasia is typically classified into four types according to Dejour et al. [2]. This classification considers the depth and shape of the trochlear groove as a crucial factor for stabilizing the patella primarily in the first 30° of flexion. Different trochleoplasties have been described trying to address this problem [3]. Out of these, the Dejour deepening trochleoplasty [4] and the subchondral deepening trochleoplasty (Bereiter technique) [5] have been favoured and modified [6]. Although the results of such trochleoplasties are satisfying, they receive substantial criticism because of the high surgical complexity and the high rate of complications [3], and hence their indication is restricted. Most surgeons only use trochleoplasties in severe dysplasia or as a second line treatment after previous failed surgery [7].

Dejour et al. described the different types of trochlear dysplasia and listed major and minor instability factors [2]. According to this approach, the ‘menu à la carte’ was introduced by the Lyon group to address the individual needs of each patient [8]. However, the length of the lateral trochlea was not included as evaluation parameter of patellofemoral disorders. Therefore, another type of trochlear dysplasia not included in the classification of Dejour et al. [2] has been described: a dysplasia defined by a short lateral articular trochlea quantified by the trochlear index [9]. A short lateral articular trochlea leads to failure in stabilizing the patella close to full extension of the knee [9]. To address this type of pathology, a trochlear lengthening osteotomy has been described [10]. To the best of our knowledge, to date only one report involving two cases reported the clinical outcome of this new osteotomy [11].

The purpose of the present study was to describe the technical aspects as well as to evaluate the functional and clinical outcome of a lengthening osteotomy of the lateral trochlea ridge as an additional or isolated procedure in patients with patellofemoral pain and/or patella instability, presence of a trochlear dysplasia Dejour type A or lack of Dejour type dysplasia and short lateral articular trochlea quantified by the lateral condyle index.

## Materials and methods

This study was approved by the regional ethics board.

## Inclusion/indications of the lateral trochlear lengthening osteotomy

**Fig. 1:**
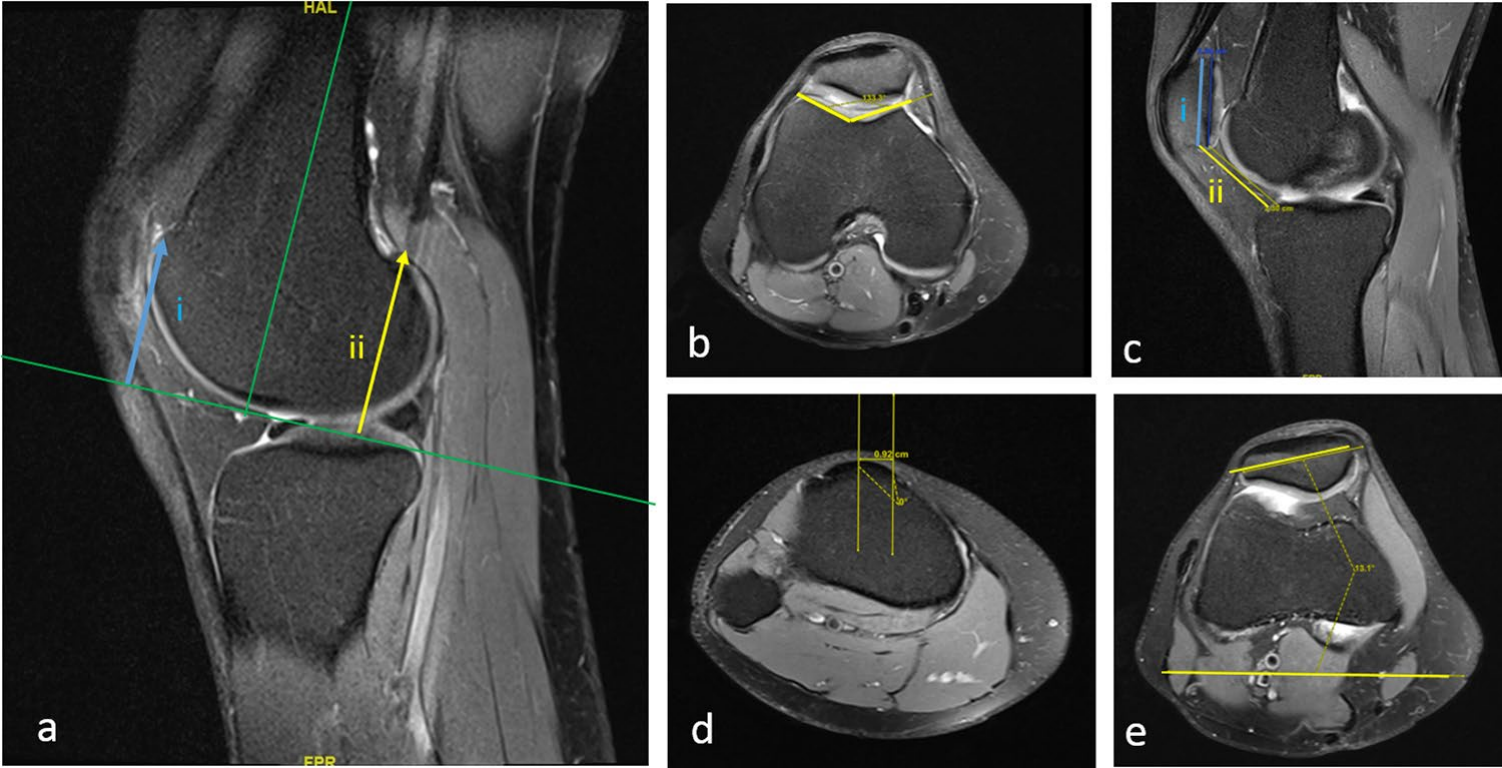
Indication for a lateral lengthening osteotomy in a patient with short lateral trochlea (lateral condylar index i/ii = 84%, **a**) and lack of trochlear dysplasia Dejour type (sulcus angle 133°, **b**), normal patellar height quantified by a normal Caton–Deschamps index of 0.95 (i/ii, **c**), TT-TG distance of 9.2 mm (**d**), and normal patellar tilt of 13° (**e**)

Over a period of 3 years, six consecutive patients with an indication for a lateral trochlear lengthening osteotomy were operated by two senior surgeons and included in this study. The indications for the lateral trochlea lengthening osteotomy included the presence of patellofemoral pain and/or patella instability with a short lateral condyle as quantified by the lateral condylar index (values below 93% are considered pathological) [9] and an absence of other clinical or radiological abnormalities to be corrected according to the Lyon ‘menu à la carte’. Namely, this procedure was indicated in patients with trochlear dysplasia Dejour type A or lack of Dejour type dysplasia, normal tibial tubercle to trochlear groove (TT-TG) distance (< 20 mm)[1], Caton–Deschamps index (0.6–1.2) [12], patellar tilt (< 20°) [1] and lack of femoral and tibial torsional deformities (Fig. 1).

### Pre- and postoperative clinical and radiological measurements

**Fig. 2:**
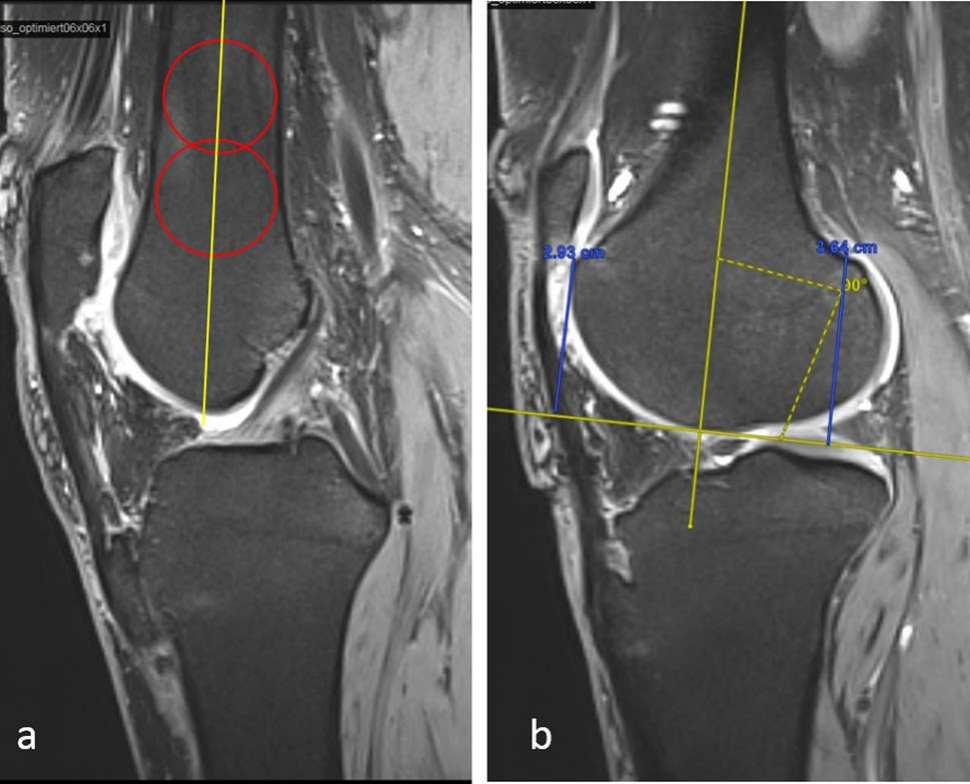
The lateral condylar index: two circles are drawn in a sagittal MRI image at the level where the anterior cruciate ligament is present at its entire length. The line connecting the centers of the two circles defines the femoral axis. A line vertical to the femoral axis tangential to the distal femoral cartilage serves as baseline to measure the length of the anterior (*a*) and posterior (*p*) cartilage length. The value (*a*/*p* × 100%) is the lateral condylar index. In this patient a pathologic lateral condylar index of 80% was calculated

Preoperatively, patients were evaluated clinically and radiologically. Clinical evaluation included range of motion (ROM), lateral gliding test [13], patellar apprehension test (Fairbank’s test) [14] and moving patellar apprehension test (MPAT) [15] as well as control for torsional deformities of the tibia [16] and of the femur [17]. The radiological evaluation included anteroposterior and lateral plane radiographs of the knee, skyline patellar view images and magnetic resonance images (MRI). Torsional deformities were investigated with MRI and computer tomography (CT) scans only if the patient had pathologic values on the clinical examination of the femoral and tibial torsion. The trochlea dysplasia according to the Dejour classification was documented according to the axial views in the MRI [2, 18]. Furthermore, in sagittal MRIs the Caton-Deschamps index [19] was measured to evaluate a possible patella alta. In axial MRIs the TT-TG distance [20] was measured to evaluate a possible tubercle lateralization and the patellar tilt [1] as a sign of muscular and ligamentous balance. The presence of a Biedert type trochlear dysplasia was evaluated according to the lateral condylar index. The measurement of this index has been previously described [9]. Briefly, sagittal MRIs are used to draw the axis of the femur at the level where the anterior cruciate ligament is present at its entire length. The length of the most anterior and posterior aspects of the cartilaginous part of the lateral condyle is measured. The index of the anterior/posterior cartilaginous length is the lateral condylar index, expressed as a percentage (Fig. 2). Values below 93% are considered pathologic, and values below 86% are diagnostic for the presence of a short lateral condyles.

Patients were followed up 6 weeks, 12 weeks, 6, 12, and 24 months postoperatively. An additional last follow up was performed at the time of conduction of this study. The initial clinical tests were performed at each follow up. Anteroposterior and lateral views of the knee as well as axial patellar views were performed 6 weeks postoperatively. Additionally, an MRI was performed in patients having discomfort at the 12 weeks follow up. The subjective evaluation was conducted through the Kujala Anterior Knee Pain Scale [21], the Lysholm Knee Score and the Tegner Activity Score [22]. Furthermore, patients were asked to subjectively evaluate the outcome of the operation according to the following options (subjective evaluation score): 5—excellent; 4—somewhat improved; 3—unchanged; 2—somewhat worse; 1—significantly worse [23], and asked if they would have the operation again. Lastly, isokinetic knee flexor and extensor strength tests were performed at 60°/s using an isokinetic dynamometer (Biodex System 4 Pro: Biodex Medical Systems, Shirley, NY, USA). The limb symmetry index (LSI) [24] (maximum torque operated side/maximum torque unaffected side × 100) of the flexors (hamstrings) and extensors (quadriceps) as well as the hamstring to quadriceps ratio (*H*/*Q* ratio) [25] were calculated.

### Procedure

**Fig. 3:**
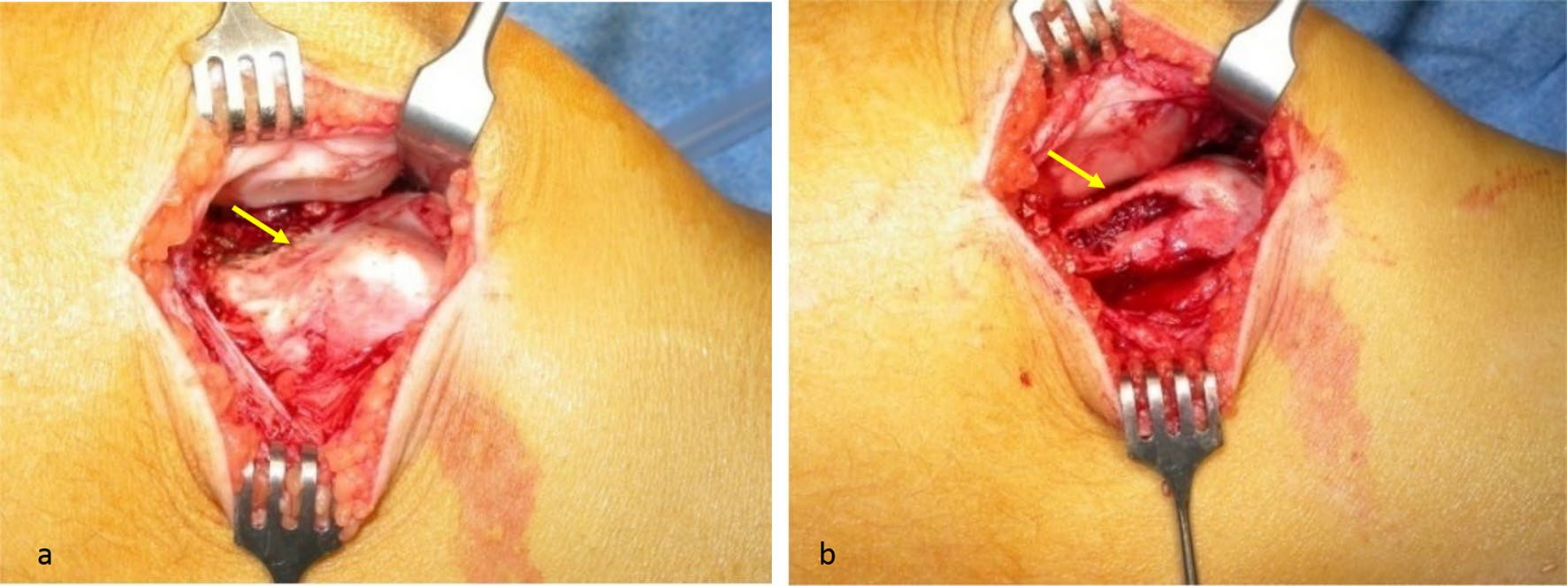
Intraoperative images of a lateral lengthening osteotomy. Note the short lateral articular trochlea before the intervention (arrow, **a**) and the lengthening of the lateral trochlea after the intervention (arrow, **b**)

**Fig. 4:**
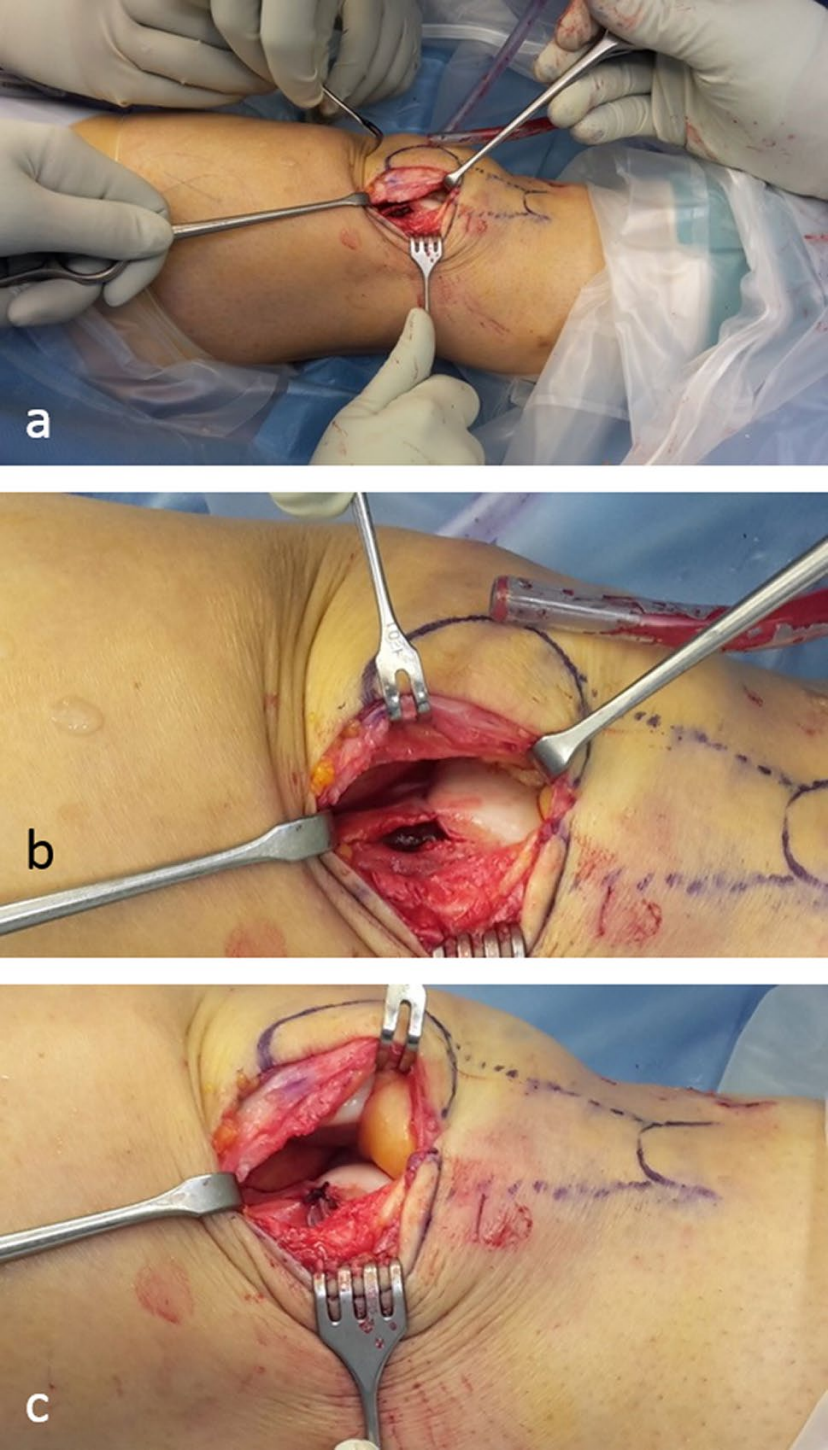
Intraoperative images of a lateral lengthening osteotomy. Note the short incision (**a**), the opening of the osteotomy and the filling with cancellous bone (**b**) as well as the suturing of the periosteum (**c**)

**Fig. 5:**
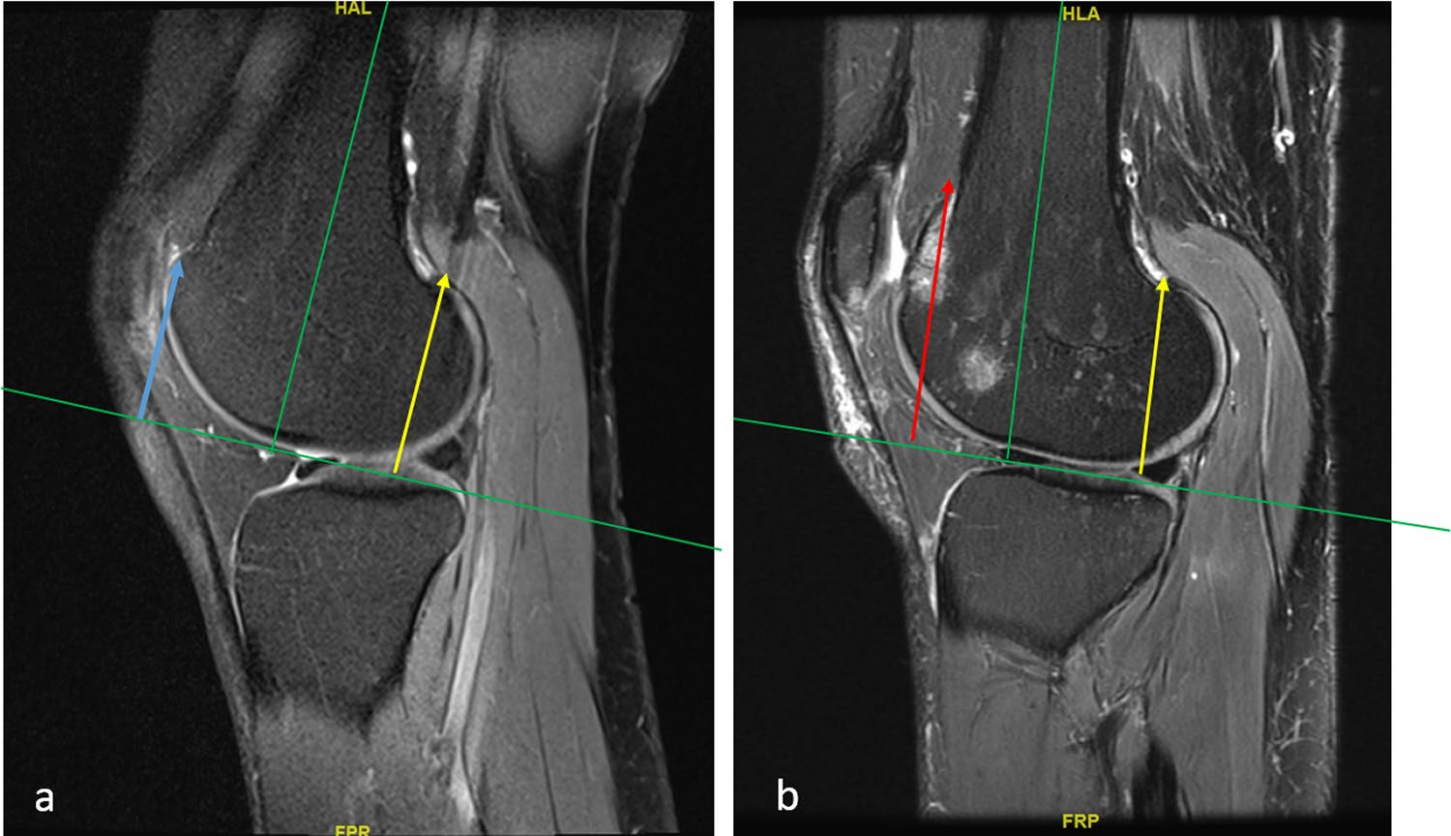
Preoperative (**a**) and postoperative (**b**) MRI images of a patient with a lateral trochlear lengthening osteotomy. Note the difference between the anterior condyle length before the operation (blue arrow, **a**) and after the operation (red arrow, **b**). The lateral condylar index was 84% preoperatively and 120% postoperatively. The yellow arrow represents the posterior condyle length and the green line the femoral axis (**a**, **b**)

**Fig. 6:**
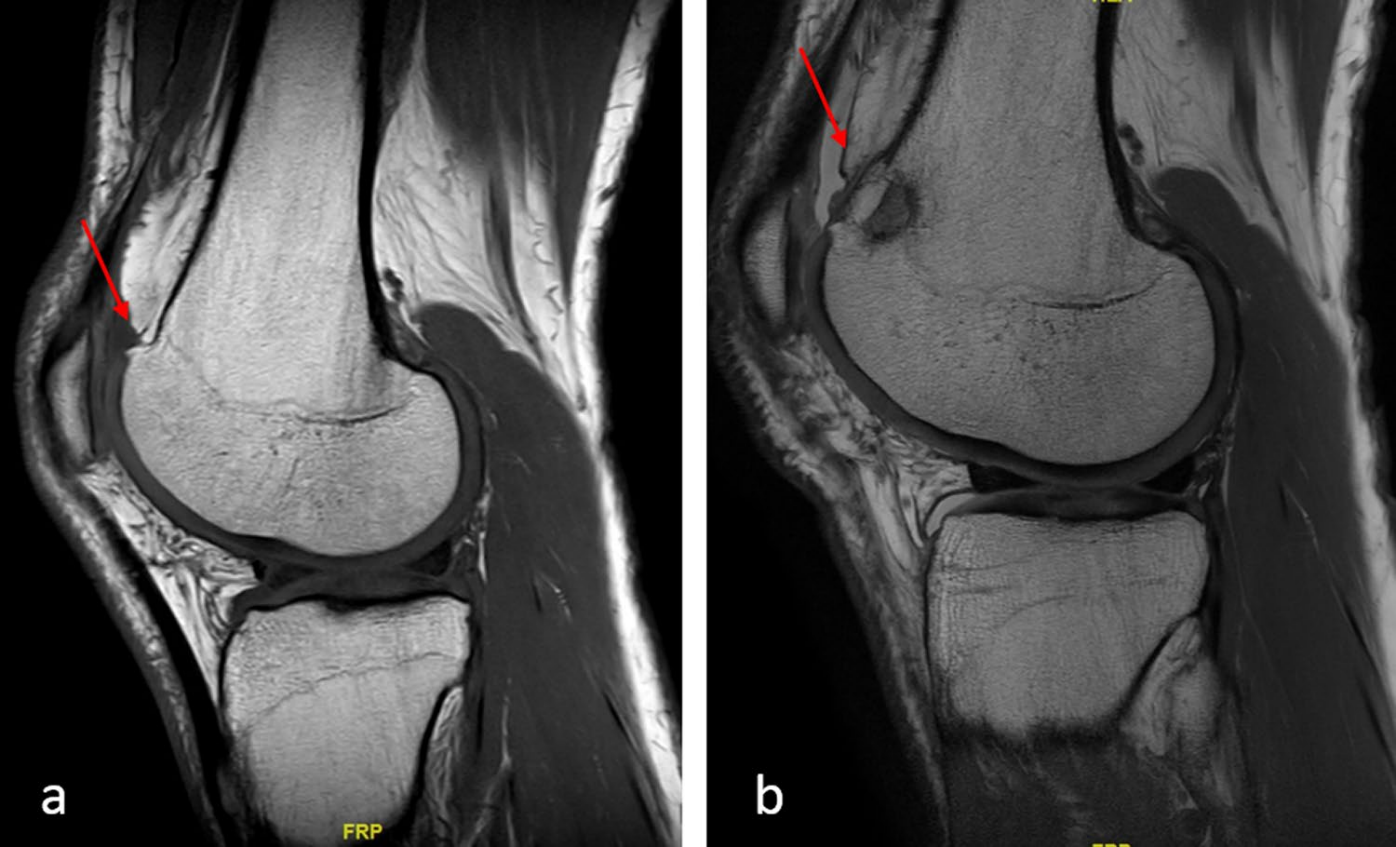
Preoperative (**a**) and postoperative (**b**) MRI images of a lateral trochlear lengthening osteotomy in a patient with short lateral trochlea and presence of a lateral bump (red arrow, **a**). Note the lengthening of the trochlea and the leveling of the bump postoperatively (red arrow, **b**)

**Fig. 7:**
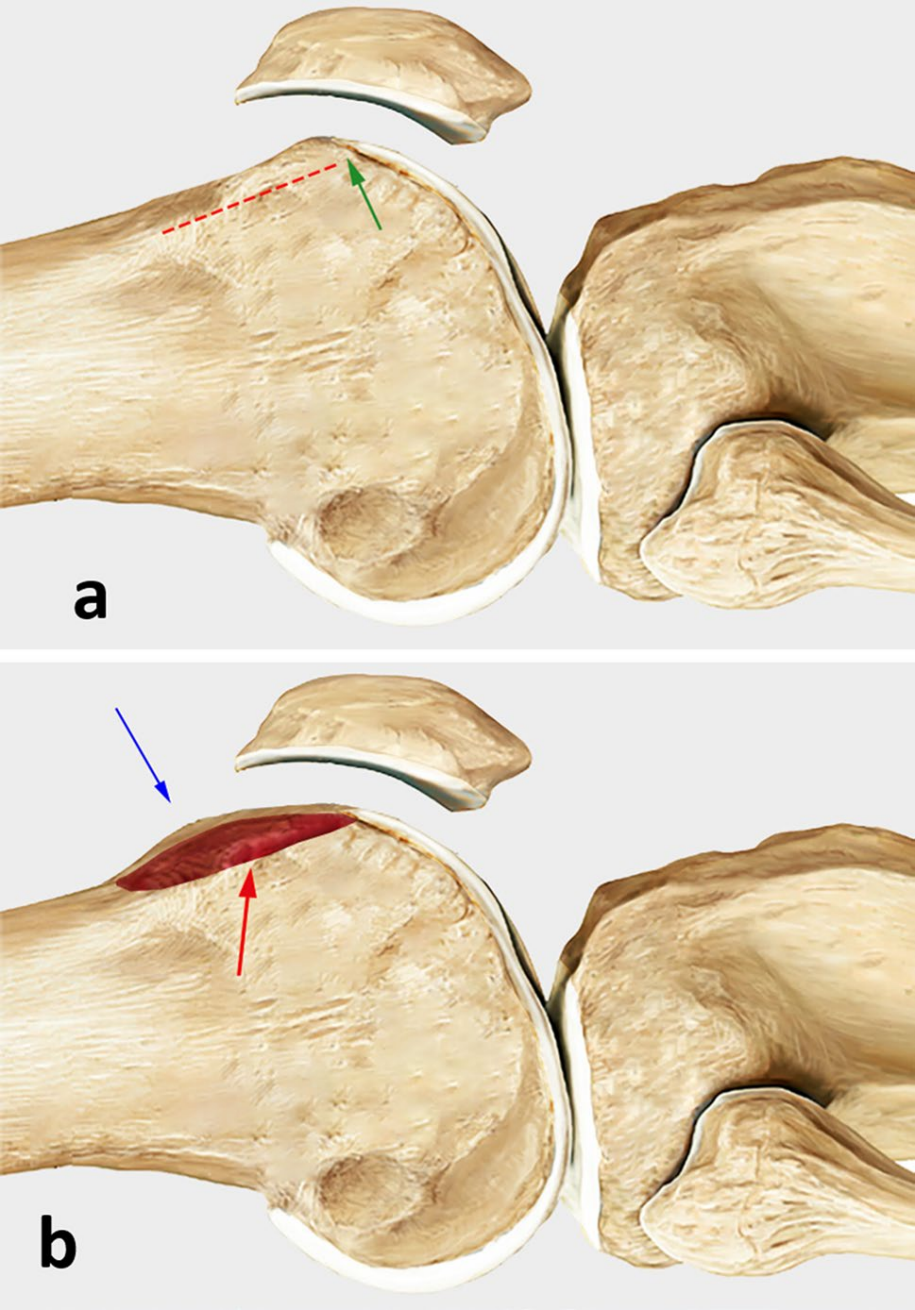
**a** An osteotomy was performed with a chisel 5 mm posterior to the lateral cartilage of the trochlea along the anteroposterior axis (red dashed line), beginning at the level of trochlear cartilage to periosteum transition (green arrow) in craniocaudal direction. **b** The cartilage and periosteum were elevated proximally providing a lengthening of the lateral trochlea (blue arrow). Cancellous bone was harvested from the lateral femoral condyles and inserted in the osteotomy gap in a press fit technique (red arrow)

The patients were operated according to the technique described by Biedert et al. [10]. Through a short lateral parapatellar approach the length of the lateral trochlea was first observed, and the pathology confirmed. An osteotomy was performed with a chisel 5 mm posterior to the lateral cartilage of the trochlea along the anteroposterior axis and at the level of trochlear cartilage to periosteum transition in craniocaudal direction. The cartilage and periosteum were elevated proximally. Cancellous bone was harvested from the lateral femoral condyles and inserted in the osteotomy gap in a press fit technique. Allograft was used in case that the harvested cancellous bone was not sufficient. The periosteum was reattached with an absorbable Vicryl size 2 suture (Figs. 3, 4, 5). In case of the presence of a symptomatic lateral bump this was corrected as part of the lengthening procedure (Fig. 6). Figure 7 demonstrates an illustration of the technical procedure.

### Postoperative care

Since the lateral lengthening osteotomy is rarely performed as a single operation, a part of the rehabilitation may be dictated by the concomitant procedures. Postoperatively, the patients were mobilized with partial weight bearing of 15 kg for 6 weeks. A hinged knee brace with a restriction of the knee joint ROM of 0/0/60° was applied for 3 weeks and 0/0/90° for another 3 weeks. Physiotherapy was initiated at the first postoperative day and continued for 6 months postoperatively. For the first 6 weeks only limited exercises with the goal of effusion elimination, gait control and leg control restoration were performed. After week 6, continuous passive motion and active ROM exercises, closed chain quadriceps exercises, and balance and proprioception exercises were included. After week 12, sport specific exercises were added. Return to sports was allowed when full ROM and lack of discomfort were achieved.

## Results

Four of the six patients had a patellar instability and two had a lateral patellar compression syndrome. Three had chondral lesions grade III or IV according to International Cartilage Repair Society (ICRS) Cartilage Lesion Classification System [26]. Five patients had been previously operated at another institution before being referred to our institution. Concomitant procedures at our institution included interventions at the medial patellofemoral ligament (MPFL) in five cases and cartilage procedures in three cases. The diagnosis, procedures prior to the presentation at our institution, as well as the concomitant procedures are presented in Table 1. The radiological parameters of each patient are presented in Table 2. One patient was lost to follow up. One patient was followed up until 1 year postoperatively but did not present herself at the 2-year follow up because of her remote place of residence. However, she reported excellent results during the telephone interview 2 years postoperatively. All other patients attended the final follow up a minimum of 2 years postoperatively. One patient had to be revised at an external institution because of an overtight MPFL reconstruction. There were no other intra- or postoperative complications. No patient had a postoperative dislocation or signs of instability. The lateral gliding test [13], the patellar apprehension test (Fairbank’s test) [14] and the MPAT [15] were negative for all patients at each follow up, and the knee flexion was above 120° with no extension deficit at the last follow up. The clinical scores of each patient at the last follow up can be found in Table 3. Two patients had full strength recovery compared to the contralateral side as well as a satisfactory *H*/*Q* ratio while two had significant strength deficits and pathologic *H*/*Q* values (Table 3).

**Table 1:** Patients who received a lateral trochlear lengthening osteotomy

Patient ID	Sex, age (years)	Diagnosis	Number of patellar dislocations	Procedures prior to the presentation in our clinic	Concomitant procedures
1	F, 34	Patellar instability	1	None	MPFL reconstruction with gracilis autograft
2	F, 47	Patellar chondral lesion grade IV, Lateral patellar compression syndrome	0	1. Medialization and distalization of the tibial tubercle 2. Arthroscopic debridement of the chondral lesion	MPFL reconstruction with semitendinosus autograft, lateral patellar retinacular lengthening, microfracturing
3	F, 31	Patella instability Patellar chondral lesion grade IV	2	Distalization of the tibial tubercle	MPFL reconstruction with gracilis autograft, Autologous matrix induced chondrogenesis (AMIC)
4	M, 45	Unstable chondral fragment Lateral patellar compression syndrome	0	Autologous chondrocyte implantation retropatellar	Refixation of chondral fragment
5	M, 27	Patellar instability	> 3	Knee arthroscopy, debridement	MPFL reconstruction with gracilis autograft
6	F, 23	Patellar instability Patellar and trochlear chondral lesion grade III	> 3	MPFL reconstruction with semitendinosus autograft	MPFL revision and fixation at anatomical femoral origin, chondroplasty with chondrofiller liquid trochlea and patella

Preoperative diagnosis, number of patellar dislocations preoperatively, number a type of procedures prior to presentation in our clinic as well as concomitant procedures are described

*MPFL* medial patellofemoral ligament, *F* female, *M* male

**Table 2:** Radiological measurements of patients treated with a lateral trochlea lengthening osteotomy

Patient ID	Trochlea dysplasia (Dejour classification)	Caton–Deschamps index	TT–TG (mm)	Patellar tilt (°)	Trochlea dysplasia Biedert type	Lateral condylar Index (%)
1	No dysplasia	0.95	9.2	13	Yes	84
2	A	1.16	11.3	17	Yes	80
3	A	1.15	5.1	6	Yes	90
4	No dysplasia	1.01	11.9	4	Yes	82
5	A	1.12	12.1	19	Yes	74
6	A	1.14	15.6	32	Yes	82

*TT–TG* tibial tubercle to trochlear groove distance

**Table 3:** Clinical scores and results of the strength measurement at the final follow up

Patient ID	Last follow up (months)	Lysholm score postoperative (preoperative)	Tegner activity score postoperative (preoperative)	Kujala score postoperative	Subjective evaluation Score (Would you have surgery again?)	LSI (quadriceps)	LSI (hamstrings)	*H*/*Q* ratio affected side	*H*/*Q* ratio unaffected side
1	53	76 (79)	6 (6)	95	5 (yes)	101.7	105.4	60.3	58.2
2	65	100 (37)	5 (2)	96	5 (yes)	96.9	87.1	53.5	60.0
3	57	85 (45)	5 (3)	75	5 (yes)	80.5	89.0	97.8	88.4
4	24	95 (60)	7 (5)	94	5 (yes)	45.8	108.6	131.0	55.2
5	3	n.a	n.a	n.a	n.a	n.a	n.a	n.a	n.a
6	24^a^	n.a	5 (2)	n.a	5 (yes)	n.a	n.a	n.a	n.a

LSI (limb symmetry index)—maximal torque of the operated side/maximal torque of the unaffected side × 100 (values between 90 and 110% are considered normal); *H*/*Q* ratio (hamstrings/quadriceps ratio)—maximal torque of the hamstrings/maximal torque of the quadriceps (values from 55 to 65 are considered normal)

^a^Follow up at 24 months was performed by telephone interview

## Discussion

This study presents the surgical procedure and functional outcomes of the lateral trochlea lengthening osteotomy. The outcomes of this case series allow us to recommend this procedure for selected patients. This is highly relevant for orthopaedic surgeons because it presents an additional treatment option in patients lacking the indication criteria for other surgical options.

The presence of a short lateral trochlea and the procedure of a lateral trochlear lengthening osteotomy are not included in the diagnostic and treatment concepts of most orthopaedic surgeons. A recent systematic review on trochleoplasties did not include this procedure as possible treatment [3]. Furthermore, Ntagiopoulos et al. [7] concluded that the ideal indication for a trochleoplasty is a presence of a high grade trochlear dysplasia (B or D), and Camathias et al. [6]—although including the lateral condylar index in their diagnostic procedures—considered a trochlea dysplasia type A as a contraindication for a trochleoplasty.

The ‘menu à la carte’ as introduced by the Lyon group [8] provides individual strategies tailored to the needs of each patient based on the original works of Dejour et al. [1, 2, 7]. This algorithm distinguishes between four major pillars causing patellar instability that can be addressed individually. Namely, a lateral tibial tubercle can be addressed with a medialization osteotomy; a patella alta with a distalisation of the tibial tubercle; a pathological patellar tilt with ligament balancing procedures and a trochlea dysplasia with a trochleoplasty. Nevertheless, as in this case series, there are rarely patients with patellofemoral pain and/or instability that do not match any of these pathologies. If the lateral condylar index is not controlled and the option of a lengthening osteotomy not discussed, these patients could remain untreated or be treated with unnecessary surgeries. In this case series, five of six patients were already unsuccessfully operated with different techniques before presenting at our clinic (Table 2). This could at least partially explain the large residual strength deficits presented in the patients of the present study because they were inactive for a long time prior to the operation. Postoperatively, the patients had good functional outcomes, lack of postoperative dislocations and were satisfied with the result of the operation. There were significant strength deficits and pathologic H/Q values in two patients. Although it is difficult to identify the reason of these deficits, it seems logical that it is the result of the long period of symptoms of the patients prior to being referred to our institution rather than with the operation itself.

In our experience, the lateral trochlea lengthening osteotomy is rarely indicated as solitary procedure. An isolated trochlea lengthening osteotomy would be indicated in patients with patellofemoral pain syndrome and short lateral trochlea without patella instability, further pathologies according to the ‘menu à la carte’ or chondral lesions. This was not the case in any of our patients. In the present study all patients received a concomitant procedure; five of the six patients received a concomitant MPFL reconstruction. Furthermore, the patient group being treated with this operation was very heterogeneous. This makes drawing conclusions regarding the single effect of this procedure difficult. Nonetheless, this procedure involves a limited surgical approach and—in contrast with other trochleoplasties—does not violate the articular surface, hence having minimal or no impact on the cartilage. Therefore, it is not expected to be associated with an increased risk of osteoarthritis. The theoretical risk of a cartilage fracture during the performance of the osteotomy did not appear in our case series. Moreover, according to Biedert et al. [10], even if this complication occurs it should be of no importance as long as the sharp edges are smoothed. Furthermore, this procedure only requires the removal of a limited amount of cancellous bone from the lateral condyle without further incisions and does not require any hardware. Altogether, we believe that this procedure provides an excellent choice as a safe, less invasive, complimentary procedure in selected patients. In case of a concomitant MPFL reconstruction, the lengthening osteotomy can provide the necessary static stability to protect the MPFL reconstruction (cases 1–3, 5–6). In case of a presence of a bony bump, the trochlear lengthening with a cartilage repair as concomitant operation and hence bump levelling may improve patellofemoral biomechanics and provide the necessary bony congruence to allow cartilage healing (cases 2–4, Fig. 6).

### Strengths and limitations

The small sample size, the heterogeneity of the patients and the lack of control group are major limitations of this study. However, because this procedure is performed rarely and most patients had prior surgery, it would be unrealistic to expect a controlled study with a large sample size on this topic in the near future. Furthermore, the operation was not performed as a single intervention and hence it is not possible to evaluate its solitary effect.

## Conclusion

We recommend measuring the lateral condylar index and considering the indication of a lateral trochlear lengthening osteotomy as an additional or isolated procedure in selected patients with trochlear dysplasia Dejour type A or lack of dysplasia and short lateral articular trochlea depending on the extent of the patellar instability.

## Abbreviations

LSI: Limb symmetry index
*H*/*Q* ratio: Hamstrings/quadriceps ratio
TT–TG distance: Tibial tubercle–trochlear groove distance
